# Genetic variation at the *CYP2C19* gene associated with metabolic syndrome susceptibility in a South Portuguese population: results from the pilot study of the European Health Examination Survey in Portugal

**DOI:** 10.1186/1758-5996-6-23

**Published:** 2014-02-18

**Authors:** Vânia Gaio, Baltazar Nunes, Aida Fernandes, Francisco Mendonça, Filomena Horta Correia, Álvaro Beleza, Ana Paula Gil, Mafalda Bourbon, Astrid Vicente, Carlos Matias Dias, Marta Barreto da Silva

**Affiliations:** 1 Departamento de Epidemiologia, Instituto Nacional de Saúde Doutor Ricardo Jorge, Lisboa, Portugal; 2 Laboratório de Saúde Pública Dra. Laura Ayres, Faro, Portugal; 3 Administração Regional de Saúde do Algarve, Faro, Portugal; 4 Departamento de Promoção da Saúde e Prevenção das Doenças Não Transmissíveis, Instituto Nacional de Saúde Doutor Ricardo Jorge, Lisboa, Portugal

**Keywords:** Metabolic syndrome, *CYP2C19* gene, Genetic association study, Continuous MetS score

## Abstract

**Background:**

Metabolic syndrome (MetS) is a cluster of conditions that occur together, increasing the risk of heart disease, stroke and diabetes. Since pathways implicated in different diseases reveal surprising insights into shared genetic bases underlying apparently unrelated traits, we hypothesize that there are common genetic components involved in the clustering of MetS traits. With the aim of identifying these common genetic components, we have performed a genetic association study by integrating MetS traits in a continuous MetS score.

**Methods:**

A cross-sectional study developed in the context of the Portuguese Component of the European Health Examination Survey (EHES) was used. Data was collected through a detailed questionnaire and physical examination. Blood samples were collected and biochemical analyses were performed. Waist circumference, blood pressure, glucose, triglycerides and high density lipoprotein cholesterol (HDL) levels were used to compute a continuous MetS score, obtained by Principal Component Analysis. A total of 37 single nucleotide polymorphisms (SNPs) were genotyped and individually tested for association with the score, adjusting for confounding variables.

**Results:**

A total of 206 individuals were studied. Calculated MetS score increased progressively with increasing number of risk factors (*P* < 0.001). We found a significant association between *CYP2C19 rs4244285* and the MetS score not detected using the MetS dichotomic approach. Individuals with the A allelic variant seem to be protected against MetS, displaying a lower MetS score (Mean difference: 0.847; 95%CI: 0.163-1.531; *P* = 0.015), after adjustment for age, gender, smoking status, excessive alcohol consumption and physical inactivity. An additive genetic effect of *GABRA2 rs279871*, *NPY rs16147* and *TPMT rs1142345* in the MetS score variation was also found.

**Conclusions:**

This is the first report of a genetic association study using a continuous MetS score. The significant association found between the *CYP2C19* polymorphism and the MetS score but not with the individual associated traits, emphasizes the importance of lipid metabolism in a MetS common etiological pathway and consequently on the clustering of different cardiovascular risk factors. Despite the sample size limitation of our study, this strategy can be useful to find genetic factors involved in the etiology of other disorders that are defined in a dichotomized way.

## Background

Metabolic syndrome (MetS) is a cluster of conditions — increased blood pressure, high blood glucose level, excess body fat around the waist and abnormal cholesterol levels — that occur together. It is strongly associated with cardiovascular diseases (CVD) and Type 2 Diabetes, increasing the risk of developing these disorders 2 and 5 fold, respectively [1]. MetS incidence and prevalence have clearly been rising worldwide, largely because of the increase in obesity rates, sedentary lifestyles and aging populations, and it is currently considered a significant public health problem [2]. In Portugal, the MetS prevalence, estimated for 2008, was 27.5% with regional variations, being highest in the Alentejo (30.99%) and lowest in the Algarve (24.42%) [3].

Due to the existence of multiple definitions considering different categorical cut-points, a consensus definition for MetS clinical diagnosis has been recently proposed. According to this definition, MetS is diagnosed when there are present at least three of the following five MetS features: abdominal obesity, elevated blood pressure, dyslipidemia (elevated triglycerides and low levels of high-density lipoprotein cholesterol), and hyperglycemia. Medication for any of these features is also considered as an indicator in the criteria for clinical diagnosis of MetS [4].

As a complex condition, MetS results from a complex interplay between many genetic and environmental factors. Lifestyle risk factors, particularly caloric excess diet and physical inactivity, seem to play an important role in MetS condition [5] but there is also evidence that its traits are highly heritable [6]. In a recent review of genome-wide association studies (GWAS), most of the single nucleotide polymorphisms (SNPs) associated with MetS traits are SNPs involved in lipid metabolism, like *FTO* rs9939609, *TCF7L2* rs7903146, *IL6* rs1800795, *APOA5* rs662799, *APOC3* rs2854117 and *CETP* rs708272 [7]. However, the identified variations explain only a very small fraction of disease burden in the population at large, suggesting that other genetic variants and interacting environmental factors are contributing to MetS susceptibility. Another important issue is that GWAS have identified numerous *loci* influencing metabolic risk traits individually, but to date, no *loci* have been found affecting the entire spectrum of MetS traits [8,9]. These limitations may be due to the highly heterogeneous groups originated by the traditional dichotomic MetS approach, reflecting associations with particular individual traits. In this context, and given that comparison of pathways and processes implicated in different diseases are revealing surprising insights into the shared genetic bases underlying apparently unrelated traits [10], our main hypothesis is that there is a common genetic component underlying the clustering of MetS traits.

Although the consensus dichotomized definition previously described remains useful for clinical practice, it loses statistical power and information when performing association studies. There are multiple evidences that a continuous outcome increases the statistical power in genetic association studies instead of a dichotomous phenotype [8], and consequently for genetic epidemiological approaches, a continuous MetS score, obtained by integrating all MetS traits, would be a more appropriate and valid alternative to study the underlying risk factors responsible for that condition [11]. Therefore, taking these issues into account, the purpose of this study was to identify genetic factors associated with MetS, using a Principal Component Analysis (PCA) derived continuous MetS score, which has been previously validated [11], to perform a genetic association study using SNPs in candidate genes related to MetS features, like glucose/insulin homeostasis, cardiovascular regulation, body mass index and lipid/drug metabolism.

## Methods

### Study design and participants

We have performed a cross-sectional study as designed for the pilot study of the Portuguese Component of the European Health Examination Survey (EHES) project [12,13]. This pilot study was conducted between 2010 and 2011, in the population covered by the São Brás de Alportel (Algarve) Health Center, constituted by 11089 individuals (2.6% of the total Algarve population). It consisted on an observational and descriptive epidemiological study with data collected through a detailed questionnaire (including socio-demographic factors, health state and health determinants) and physical examination. A blood sample for further biochemical analysis was also collected. Participants were selected using a simple random sampling scheme from the National Health System card number database, which covers over 99% of the total population from the São Brás de Alportel Health Center users. All participants were given a brief description of the objectives of the study, after which they signed an informed consent form. The study protocol was approved by the Ethics Committee of National Health Institute Doctor Ricardo Jorge and by the National Commission for Data Protection.

### Measurements and blood sample collection

Blood pressure, anthropometric (weigh, height and waist circumference) and biochemical (high density lipoprotein cholesterol (HDL), triglycerides (TG) and glucose) measurements were performed in accordance with the recommendations proposed by FEHES – “*Feasibility of a European Health Examination Survey*” [14]. Genomic DNA was isolated from whole blood containing EDTA according to standard methods [15].

### Calculation of the continuous MetS score

The MetS score was calculated by principal component analysis (PCA) with varimax rotation, as previously described [11]. We have considered six quantitative MetS risk factors (waist circumference, diastolic blood pressure (DBP), systolic blood pressure (SBP), glucose, TG and HDL plasma levels) assuming the newly harmonized diagnostic criteria of the MetS [4].

Shapiro-Wilk test was used to assess the normality of the MetS quantitative risk factors. Those non-normal distributed were normalized (SBP, Glucose, HDL and TG were normalized using log_10_[log_10_(SBP)], 1/[log_10_(Glucose)]^10^, [ln(HDL)]^2^ and log_10_[log_10_(TG)] respectively). We have used the first and second principle components (PC) which represent large fractions of MetS variance (considering eigenvalues >1.0). A final MetS score was computed by summing the first two individual PC scores, weighted by the relative contribution of each principal component in the explained variance. A higher MetS score indicates a less favorable MetS profile.

Validity of the MetS score was tested using the ANOVA for trend analysis. T-Test and ANOVA were also used to test mean difference values of the MetS score between groups of risk factor categories. The consensus definition recently proposed for MetS clinical diagnosis [4] was used to categorize individuals. According to this definition, MetS is diagnosed when there are present at least three of the following five MetS features: elevated waist circumference (men ≥94 cm, women ≥80 cm); low HDL cholesterol (men < 40 mg/dL, women < 50 mg/dL); elevated TG (≥150 mg/dL); elevated BP (≥130/85 mmHg); and elevated glucose levels (≥100 mg/dL). Medication for any of these features is also considered as alternative indicator in the criteria for diagnosis of MetS.

### SNP selection and genotyping

To test the association between the MetS score variation and SNPs in different candidate genes potentially involved in the MetS etiology, we have selected SNPs based on their involvement in metabolic-related phenotypes. In order to capture the maximum variation with minimum genotyping effort, we have selected those that were haplotype-tagging SNPs and that represented a validated functionally relevant variation, reported in the OMIM database. Consequently, we have genotyped 37 SNPs: 13 SNPs in genes involved in glucose/insulin homeostasis *CDKAL1* rs7754840, *CDKN2A/B* rs10811661, *HHEX* rs1111875, *IGF2BP2* rs4402960, *IL6* rs1800795, *KCNJ11* rs5219, *KCNQ1* rs2237892, *MTNR1B* rs10830963, *PPARG* rs1801282, *SLC30A8* rs13266634, *TCF7L2* rs7903146, *ADCY5* rs11708067 and *KCNQ1* rs231362), 10 SNPs in genes involved in body mass index (*GNPDA2* rs10938397, *MTCH2* rs10838738, *NPC1* rs1805081, *PTER* rs10508503, *SH2B1* rs7498665, *FTO* rs9939609, *ADRB3* rs4994, *GABRA2* rs279871, *NPY* rs16147, *TMEM18* rs6548238), 7 SNPs in genes involved in cardiovascular system regulation (*ACE* rs4646994, *NOS1AP* rs12143842, *ADRB1* rs1801252, *ADRB2* rs1042714, *NOS3* rs1799983, *NOS3* rs2070744) and 7 involved in drug/lipid metabolism (*APOE* rs7412, *LDLR* rs2228671, *CYP2C8* rs10509681, *CYP2D6* rs16947, *CYP2C19* rs4244285, *TPMT* rs1142345). All SNPs were genotyped by Sequenom MassARRAY platform except for seven (*ACE* rs464699, *ADRB1* rs1801252, *ADRB2* rs1042713, *CYP2C8* rs10509681, *CYP2C9* rs1799853, *CYP2D6* rs16947 and *NOS3* rs2070744) that were genotyped by Polymerase Chain Reaction (PCR) followed by Restriction Fragment Length Polymorphism (RFLPs) analysis (Additional file 1: Tables S1 and S2).

### Statistical analysis

The statistical analysis was performed using *IBM SPSS statistics 20. P-values* < 0.05 were considered to be statistically significant. T-test and Mann-Whitney test were used to access differences of quantitative variables according to their adherence to the normal distribution, evaluated by the Shapiro-Wilk test. Proportions were compared using χ^2^ tests.

All SNPs were tested for Hardy Weinberg Equilibrium using the Hardy Weinberg R package [16], based on the χ^2^-test. The association between the MetS score and the isolated SNPs was tested by T-test. Subsequent correction for multiple comparisons was performed using the *Bonferroni* method. The association between the MetS categorized groups (participants with MetS *versus* participants without MetS) with selected SNPs was also performed using χ^2^-test. Individual association analysis between each MetS quantitative risk factor and the selected SNPs were also performed using either T-test or Mann-Whitney test, according to their adherence to the normal distribution. Lifestyle risk factors (smoking status, excessive alcohol consumption, physical inactivity and unhealthy diet) were also individually tested for association with the MetS score, using the T-test.

ANOVA for trend (*P* < 0.05) was used to assess linearity between the MetS score and the number of genetic risk factors, testing for additive genetic effects of risk variants in the increasing of MetS score values.

General linear model analysis (GLM) was used to test MetS score differences between subjects with different genotypes after adjusting for confounding variables such as age, gender, smoking status, excessive alcohol consumption, physical inactivity and unhealthy diet. Smoking status and excessive alcohol consumption were defined as previously described [17]. Inadequate physical activity was defined as practice of a regular physical activity such as running, cycling or other, in order to feel tired, less than once a week. Unhealthy diet was defined as absence of fruit or vegetables consumption on the day before the interview. Only variables contributing more than 5% for the MetS score variation were considered in the model.

## Results

### Population characteristics

From the 221 recruited participants, we have excluded those with missing blood samples or missing values for the analyzed parameters (n = 15). The final study population consisted on 206 participants, 87 (42.2%) men and 119 (57.8%) women. The participants’ age ranged from 26 to 91 years, being the mean value 56.43 ± 16.23. The general characteristics of the participants included in this study are shown in Table 1.

**Table 1:** General characteristics of the participants

	**Participants (n = 206)**
Men	42.23% (35.49-48.98%)
Women	57.77% (51.03-64.50%)
Age (years ± SD)	56.43 ± 16.23
**MetS prevalence**^**1**^	46.12% (39.31-52.92%)
MetSscore	0.00 ± 1.41
**MetS risk factors (Mean ± SD):**	
Waist circumference (cm)	95.50 ± 12.56
DBP (mmHg)	80.67 ± 9.96
SBP (mmHg)	131.72 ± 20.02
HDL (mg/dL)	53.51 ± 13.33
TG (mg/dL)	107.71 ± 60.29
Glucose (mg/dL)	103.29 ± 33.91
**MetS related diseases**	
Hypertension	26.21% (20.21-32.22%)
Type 2 Diabetes	7.3% (3.73-10.83%)
Hypercholesterolemia	12.6% (8.09-17.16%)
Total	46.12% (39.31-52.92%)
**Medication**^**2**^	43.20% (36.44-49.97%)
**Lyfestyle risk factors**	
Smokers	17.96% (12.72-23.20%)
Excessive alcohol consumption	8.74% (4.88-12.59%)
Inadequate physical activity	59.71% (53.01-66.41%)
Unhealthy diet	2.92% (29.37-42.47%)

Data is presented as mean ± standard deviation for continuous variables and % (95%CI) for proportions.

^1^For the metabolic syndrome definition, the newly harmonized diagnostic criteria was used [4].

^2^Medication for hypertension, type 2 diabetes and hypercholesterolemia was considered.

Abbreviations: *CI* Confidence interval, *MetS* metabolic syndrome, *DBP* diastolic blood pressure, *SBP* systolic blood pressure, *HDL* high density lipoprotein cholesterol, *TG* triglycerides.

**Figure 1:**
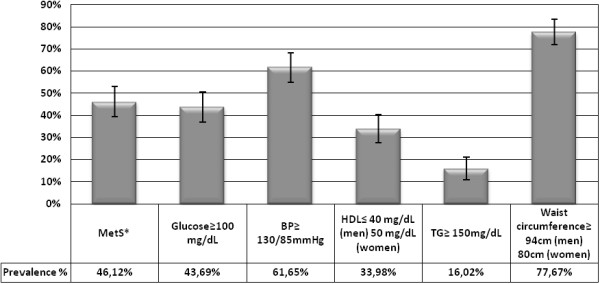
**MetS and its components prevalence.** Participants medicated for hypertension, hypercholesterolemia and diabetes were also accounted. Error bars represent the 95% confidence intervals. Abbreviations: MetS, metabolic syndrome; DBP, diastolic blood pressure; SBP, systolic blood pressure; HDL, high density lipoprotein cholesterol; TG, triglycerides. (*For the MetS prevalence calculation, the newly harmonized definition was considered [4]).

We found differences between the frequencies of auto-reported MetS related chronic diseases confirmed by the family general practitioner (Table 1) and those obtained if we considered the correspondent parameters measured in this study, although they did not reach significance. We have found 26.21% auto-reported hypertensive participants, based on information collected by the questionnaire, compared to 39.32% of participants who have values of measured blood pressure above 140/90 mmHg, the cut-off value for hypertension definition. There are also 7.3% auto-reported diabetic participants, but 17.48% of the participants have levels of glucose above 110 mg/dL, the cut-off used in pre-diabetes diagnosis. The prevalence of MetS was 46.12%, assuming the newly harmonized definition (Figure 1). The MetS components prevalences’ are present in Figure 1, taking into account the most restrictive cut-off points [4].

### Continuous MetS score calculation by PCA

In the total sample, from PCA considering the six quantitative MetS risk factors, we are able to explain 63.35% of these six components variance (PC1 and PC2 explained 35.42% and 27.43% of the variance, respectively). The measured correlations between each MetS risk factor and both principal components are presented in Table 2. BP, waist circumference and glucose levels are the components contributing more to PC1. For PC2, the main contributors are TG and HDL levels. No significant differences were found when subjects treated with medication for hypertension, hypercholesterolemia and diabetes were excluded from the analysis. In fact, medicated participants maintain altered values for the six quantitative MetS score risk factors and have higher MetS score values in comparison with non medicated participants, suggesting a medication inefficacy or absence of medication compliance.

**Table 2:** Correlation coefficients between the normalized components of MetS and the two principal components obtained from PCA

**MetS normalized components**	**Coeficient correlation**	
	**PC1**	**PC2**
Waist circumference	0.650	0.255
DBP	0.771	0.320
SBP	0.826	0.057
Glucose	-0.598	0.147
HDL	0.079	-0.885
TG	0.305	0.818

Abbreviations: *DBP* diastolic blood pressure, *SBP* systolic blood pressure, *HDL* high density lipoprotein cholesterol, *TG* triglycerides, *PC1* principal component 1, *PC2* principal component 2.

**Figure 2:**
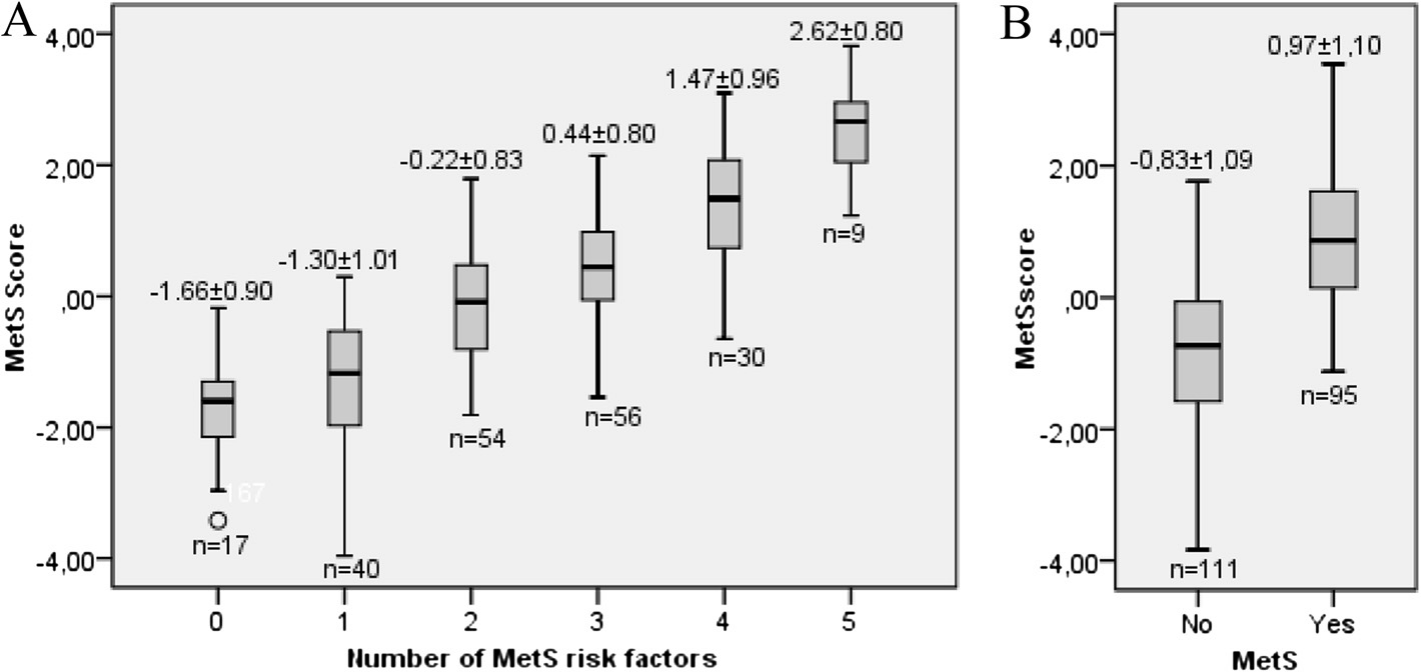
**Mets score validity. A** MetS score variation according to the number of risk factors (ANOVA for trend *P* < 0.001). **B** Comparison between the affected versus unaffected participants (T-test *P* < 0.001). The consensus MetS definition was considered to define the affection status MetS groups [4].

The MetS score adequacy and validity is shown in Figure 2A. As expected, this score increases progressively with increasing numbers of risk factors (ANOVA test for linear trend, *P* < 0.001). As observed, the MetS score is clearly higher in subjects with MetS versus subjects without (0.97 ± 1.10 versus -0.83 ± 1.09; T-test *P* <0.001), when we categorize individuals based on the dichotomic MetS definition (Figure 2B).

### Genotyping data

The descriptive statistics regarding the tested SNPs are described in Table 3. Minor allele frequencies (MAF) range from 0.027 to 0.491. No significant differences were found between the European MAF described in NCBI database [18] and the obtained MAF of the markers for the population under study. All SNPs are in Hardy-Weinberg equilibrium (*P* >0.05).

**Table 3:** List of SNPs selected in the present study

**Mechanisms**	**Gene**	**NCBI ID**	**Alteration**	**Reference**	**Control**	**Obtained**	**HWE**
					**MAF**^**1**^**(N)**	**MAF(N)**	***P-value***^***2***^
**Glucose/insulin homeostasis**	*CDKAL1*	rs7754840	C → G	[19]	0.336 (226)	0.286 (206)	0.456
	*CDKN2A/B*	rs10811661	C → T	[19]	0.199 (226)	0.201 (206)	0.056
	*HHEX*	rs1111875	A → G	[19]	0.416 (226)	0.371 (206)	0.558
	*IGF2BP2*	rs4402960	G → T	[19]	0.280 (118)	0.272 (206)	0.422
	*IL6*	rs1800795	C → G	[19]	0.465 (226)	0.337 (206)	0.917
	*KCNJ11*	rs5219	C → T	[19]	-	0.333 (206)	0.532
	*KCNQ1*	rs2237892	C → T	[19]	0.075 (226)	0.051 (206)	1.000
	*MTNR1B*	rs10830963	C → G	[20]	0.300 (120)	0.223 (206)	0.264
	*PPARG*	rs1801282	C → G	[19]	0.076 (118)	0.093 (205)	1.000
	*SLC30A8*	rs13266634	C → T	[19]	0.239 (226)	0.286 (206)	0.761
	*TCF7L2*	rs7903146	C → T	[19]	0.279 (226)	0.303 (205)	0.256
	*ADCY5*	rs11708067	A → G	[20]	0.226 (226)	0.199 (206)	0.835
	*KCNQ1*	rs231362	C → T	[19]	0.482 (112)	0.234 (128)	0.214
**Cardiovascular regulation**	*ACE*	rs4646994	Ins/Del	[21]	-	0.420 (206)	0.171
	*NOS1AP*	rs12143842	C → T	[22]	0.188 (224)	0.265 (206)	0.827
	*ADRB1*	rs1801252	A → G	[23]	-	0.108 (206)	0.711
	*ADRB2*	rs1042714	C → G	[24]	0.467 (120)	0.407 (204)	0.898
	*ADRB2*	rs1042713	A → G	[24]	0.358 (226)	0.362 (206)	0.903
	*NOS3*	rs1799983	G → T	[24]	0.342 (120)	0.417 (206)	0.072
	*LDLR*	rs2228671	C → T	[25]	0.106 (226)	0.124 (206)	1.000
	*NOS3*	rs2070744	C → T	[24]	-	0.451 (206)	0.892
**Body mass Index**	*GNPDA2*	rs10938397	A → G	[26]	0.446 (112)	0.481 (206)	0.309
	*MTCH2*	rs10838738	A → G	[26]	0.363 (226)	0.282 (206)	0.855
	*NPC1*	rs1805081	A → G	[26]	0.467 (120)	0.288 (206)	0.385
	*PTER*	rs10508503	C → T	[26]	0.092 (218)	0.075 (206)	1.000
	*SH2B1*	rs7498665	A → G	[26]	0.382 (226)	0.303 (206)	0.483
	*FTO*	rs9939609	A → T	[26]	0.449 (118)	0.361 (205)	0.396
	*ADRB3*	rs4994	C → T	[27]	0.088 (226)	0.090 (205)	1.000
	*GABRA2*	rs279871	A → G	[28]	-	0.434 (206)	0.376
	*NPY*	rs16147	A → G	[29]	0.491 (226)	0.450 (206)	0.222
	*TMEM18*	rs6548238	C → T	[26]	0.150 (220)	0.127 (205)	0.542
**Lipid/Drug metabolism**	*APOE*	rs7412	C → T	[25]	-	0.027 (161)	1.000
	*CYP2C8*	rs10509681	C → T	[30]	0.137 (226)	0.129 (206)	0.750
	*CYP2C9*	rs1799853	C → T	[30]	0.104 (106)	0.138 (206)	0.750
	*CYP2D6*	rs16947	A → G	[31]	-	0.393 (206)	0.845
	*CYP2C19*	rs4244285	G → A	[32]	0.155 (116)	0.129 (206)	1.000
	*TPMT*	rs1142345	A → G	[33]	0.027 (226)	0.032 (205)	1.000

^1^Values referring to the HAPMAP-CEU population, available in the NCBI database [18].

^2^*P-values* were obtained by “HardyWeinberg” R package, based on the χ^2^ -test.

### Association analysis

Assuming a dominance model, a significant association was found between the MetS score and the following SNPs: *CYP2C19* rs4244285 (*P* = 4.9×10^-4^), *GABRA2* rs279871 (*P* = 0.018), *NPY* rs16147 (*P* = 0.029) and *TPMT* rs1142345 (*P* = 0.003). Considering the sample size and the allelic frequency of the four significant associated SNPs: *CYP2C19* rs4244285, *GABRA2* rs279871, *NPY* rs16147, *TPMT* rs1142345, this study has 80% power to detect a mean difference of 0.642, 0.598, 0.612 and 1.132 respectively, in the MetS score variation. Regarding the *CYP2C19* rs4244285 SNP, individuals included in the GA + AA genotype group seem to be protected against MetS, displaying a lower MetS score (Mean difference: 0.792; 95%CI: 0.351-1.233; *P* < 0.001) (Table 4). After *Bonferroni* correction for multiple testing, the association between the MetS score and the *CYP2C19* rs4244285 remains significant (*P* = 0.018). Within both genotype groups, medicated individuals display higher MetS score values than non medicated ones, although not reaching significance.

**Table 4:** SNPs significantly associated with MetS score

**Gene**	**Genotype**	**n**	**MetS score**	**Mean difference**	**95%CI**	***P-value***^**a**^	**Corrected *P-value***^**b**^
***CYP2C19***	GG	156	0.192 ± 1.380	0.792	0.351-1.233	0.00049	**0.018**
rs4244285	GA + AA^1^	50	-0.600 ± 1.362				
***GABRA2***	AA	63	0.350 ± 1.374	0.504	0.087-0.921	0.018	0.670
rs279871	GA + GG^2^	143	-0.154 ± 1.409				
***NPY***	AA	58	0.342 ± 1.606	0.476	0.048-0.904	0.029	0.999
rs16147	GA + GG^3^	148	-0.134 ± 1.313				
***TPMT***	AA	192	-0.080 ± 1.375	1.199	0.413-1.984	0.003	0.109
rs1142345	GA	13	1.119 ± 1.601				

^a^T-test was used to compare MetS score mean values between the two groups.

^b^Corrected *P-values* were obtained using the *Bonferroni* test to multiple testing correction.

^1^The GA + AA group consists on 3 AA and 47 GA individuals.

^2^The GA + GG group consists on 36 AA and 107 GA individuals.

^3^The GA + GG group consists on 37 AA and 111 GA individuals.

The MetS score is presented as mean ± SD.

**Figure 3:**
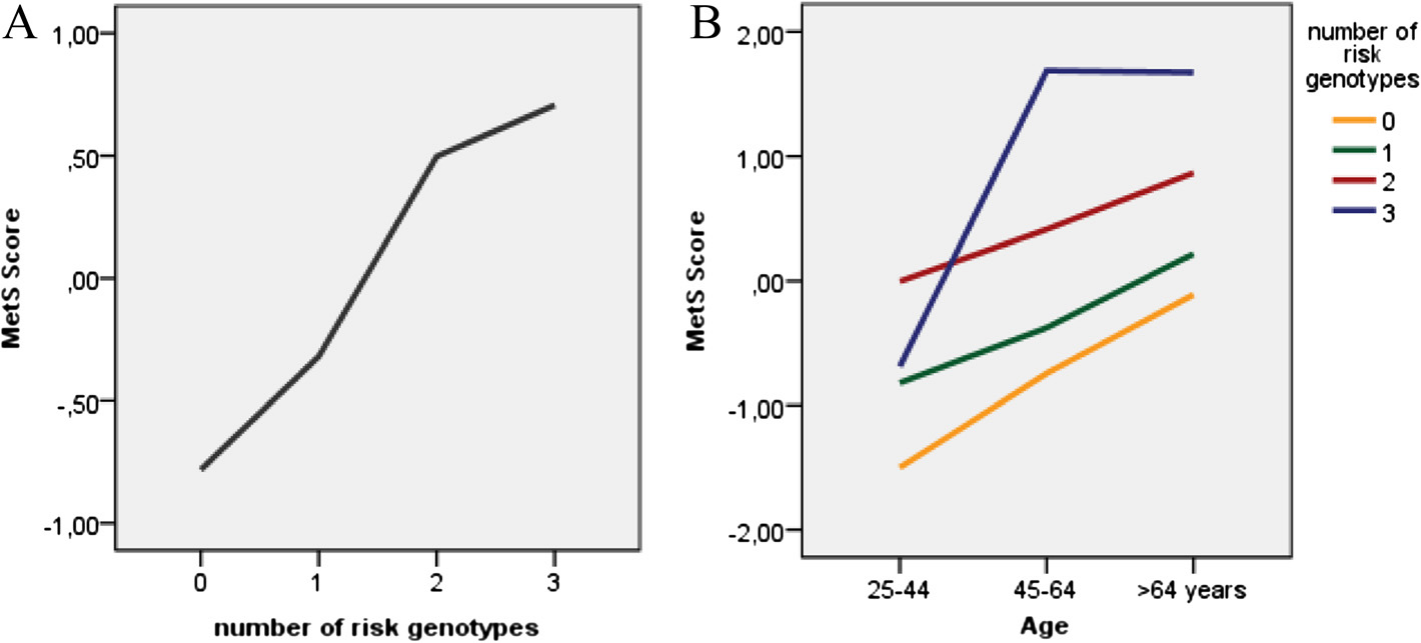
**Additive genetic effects of the SNPs (*GABRA2 *rs279871, *NPY *rs16147*, TPMT *rs1142345). A**- Influence of the number of risk genotypes in the MetS score values. MetS score increases with increasing number of genetic risk factors (ANOVA for trend *P* < 0.001). **B**- Additive genetic effect representation. Each line represents a different number of risk genotypes, considering sufficient the presence of one allele for each variant to be at risk. We have considered the 4 significantly associated SNPs previous reported: *CYP2C19* rs4244285, *GABRA2* rs279871, *NPY* rs16147 and *TPMT* rs1142345. No individuals with 4 risk genotypes for the 4 SNPs were identified in this population.

Despite the fact that associations of the three SNPs (*rs279871*, *rs16147, rs1142345*) in the *GABRA2*, *NPY* and *TPMT* genes, with the MetS score do not remain significant after multiple testing correction, we can observe an additive genetic effect of these variants in the MetS score (Figure 3A), since it increases with the growing number of genetic risk factors (ANOVA for trend *P* < 0.001). Moreover, this additive genetic effect is age independent, as shown in Figure 3B.

No association was found between the individual MetS quantitative risk factors (waist circumference, BP, glucose, TG and HDL blood levels) and the selected SNPs. Similarly, no association was found between MetS using the dichotomic definition (participants with MetS *versus* participants without MetS) and the analyzed SNPs. No association was found between the MetS score and the selected lifestyle risk factors.

### General linear model analysis

Using general linear model analysis, we found that differences on the MetS score between subjects with GG genotype and GA + AA genotype on the *CYP2C19* rs4244285 remain significant after adjustment for age, gender, smoking status, excessive alcohol consumption and physical inactivity (Mean difference: 0.847; 95%CI: 0.163-1.531; *P* = 0.015). The variable unhealthy diet was discarded since it contributes less than 5% for the MetS score variation. General linear model analysis was also performed for the other associated SNPs: *GABRA2* rs279871 (Mean difference: 0.597; 95%CI: 0.053-1.247; *P* = 0.071), *NPY* rs16147 (Mean difference: 0.854; 95%CI: 0.175-1.532; *P* = 0.014) and *TPMT* rs1142345 (Mean difference: 0.850; 95%CI: 0.086-1.787; *P* = 0.075), assuming the adjustment for the same variables.

## Discussion

GWAS have identified common variants of modest and small-effect size at hundreds of *loci* for common chronic disorders but a substantial fraction of heritability remains unexplained [34]. Given that GWAS performed on MetS traits, using a traditional dichotomic definition indicates that only a small portion of the variation in these traits can be explained by the reported common genetic variants, our aim was to identify genetic risk factors involved in MetS etiology using a quantitative phenotype. To achieve our goal we have tested the association between a group of biologically relevant SNPs in order to seek positive genetic associations with a continuous MetS score, obtained by PCA, instead of the dichotomized MetS definition. We believe that, using a continuous MetS score based on PCA to address the issue of genetic susceptibility to MetS and its clustering associated traits, will increase the statistical power and unravel the missing heritability. In fact, the MetS score based on this approach was able to explain over 63.0% of the phenotype.

In this study, we have found a significant association between the MetS score and the *CYP2C19* rs4244285. Individuals carrying the A allelic variant have a lower MetS score, suggesting that these genotypes are conferring protection against MetS. Most importantly, the differences on the MetS score between subjects with GG genotype and GA + AA genotype on the *CYP2C19* remained significant after adjustment for age, gender, smoking status, excessive alcohol consumption and physical inactivity, which are important confounding factors, suggesting a pivotal role of this gene in metabolic regulation.

Regarding the *CYP2C19* gene polymorphisms, most of the studies performed to date focus on the altered drug metabolism [35] and little importance has been given to its role on lipid metabolism involvement. It is known that the functional enzyme product of the *CYP2C19* gene also metabolizes important endogenous substrates, namely arachidonic acid, a n-6 unsaturated fatty acid, to produce epoxyeicosatrienoic (EETs) compounds which generally possess vasodilating, anti-inflammatory, anti-apoptotic, anti-thrombotic, natriurectic and cardioprotective effects [36]. On the other hand, *CYP2C19* rs4244285 consists in an aberrant splice site, generating a truncated non-functional protein, without catalytic activity [30] which has been shown to be associated with increased adverse cardiovascular events in patients medicated with clopidogrel [37]. This hypothesis is in accordance with our results, given that, within the GA + AA genotype group, medicated individuals display a higher MetS score than non medicated ones. However, further studies will be necessary to clarify whether this variant is causal or if it is in linkage disequilibrium with the true causal variant.

Given that *CYP2C19* is a highly polymorphic gene, with at least 19 allelic variants reported [38], we have to consider the potential coexistence of other variants that could compensate this defective allele by overexpressing it. Actually, a recently described gain of function allele in *CYP2C19* gene (rs12248560) is a regulatory polymorphism enhancing *CYP2C19* expression with potential to compensate the *CYP2C19* rs4244285 defective variant [39]. We expect that, with the use of next generation sequencing technologies, we will be able to further dissect genetic variation present in this gene and clarify the relative contribution of each variant to the CYP2C19 actual function.

We have also found an association between the MetS score and SNPs at the *GABRA2*, *NPY* and *TPMT* genes. Despite the fact that these associations do not remain significant after multiple testing correction, likely due to the lack of statistical power, they might represent an additive genetic effect that should be taken in consideration in the etiology of MetS. The genes *NPY* and *GABRA2* have been previously found associated with obesity and food intake, being involved in multiple central nervous system functions regulation [40-42], while *TPMT* has an important role in drug metabolism [43].

Given these results, we hypothesize that *CYP2C19* rs424428 might be involved in a common pathway, the deregulation of which, in addition to other specific genetic factors, may lead to the different MetS associated traits. This hypothesis is corroborated by the fact that, when we test associations between each MetS quantitative risk factor (waist circumference, BP, glucose, TG and HDL blood levels) and the selected candidate SNPs, no association was found. This finding suggests that there is a common basic pathway involving variants in *CYP2C19* alone or in association with other genes that may lead to the development of MetS and associated traits, similarly to what has been described for the *HLA* or *CTLA4* genes in the development of different autoimmune disorders [44].

Despite the fact that, further association and functional studies are necessary to highlight the role of *CYP2C19* in MetS etiology, we think that these results may contribute to the identification of new therapeutic targets that may be useful in the treatment of the different clustered traits instead of treating them individually. Novel therapies targeted at these newly identified genes may be developed and consequently improve the outcome of patients affected by this disorder.

## Conclusions

Our study represents an integrative approach to identifying genetic risk factors involved in MetS etiology, through a continuous MetS score obtained by PCA. This score alone explains over 63.0% of the phenotype, supporting the usefulness of a continuous MetS risk score, instead of the dichotomized MetS definition traditionally used in case-control studies.

A significant corrected association between a *CYP2C19* rs4244285 and the MetS score was clearly found. The less frequent allele of this variant seems to be conferring a protective effect to MetS susceptibility. In addition, we found that these differences remained significant after adjustment for age, gender, smoking status, excessive alcohol consumption and physical inactivity. We hypothesize that *CYP2C19* rs4244285 is involved in a common pathway, the deregulation of which, in addition to other specific genetic factors, may lead to the different MetS associated traits. This hypothesis is supported by the fact that no association was found between this SNP and each quantitative risk factor. Other variants in *GABRA2*, *NPY* and *TPMT* might represent additive genetic factors of modest effect that should be taken into consideration to understand the complete etiology of MetS, as well as environmental factors such as smoking status, diet and physical activity. Despite the need of further studies to confirm this association in a larger population with better statistical power, we are confident that these results will lead to the identification of new therapeutic targets. These may be useful in the treatment of the different clustered traits instead of treating them individually and consequently improving the outcome of patients affected by both MetS and associated chronic diseases.

## Abbreviations

BP: Blood pressure; CVD: Cardiovascular diseases; DBP: Diastolic blood pressure; EHES: European Health Examination Survey; FEHES: Feasibility of a European Health Examination Survey; GP: General practitioner; GWAS: Genome-wide association study; HDL: High density lipoprotein cholesterol; HWE: Hardy Weinberg Equilibrium; MetS: Metabolic syndrome; NCBI: National Center for Biotechnology Information; PC: Principal component; PCA: Principal component analysis; RFLPs: Restriction fragment length polymorphism; SBP: Systolic blood pressure; SNP: Single nucleotide polymorphism; TG: Triglycerides.

## Competing interests

The authors declare that they have no competing interests.

## Authors' contributions

MBS, APG and CMD performed the study design. BN, AF, FM, FHC, AB, APG, MB, AV, CMD and MBS carried out data collection. VG carried out genotyping, analyzed the data and wrote the manuscript. VG and BN conducted the statistical analysis. MBS participated in writing and editing the manuscript. BN, CMD, MBS contributed to the discussion and reviewed the manuscript. All authors read and approved the final manuscript.

## Supplementary Material

Additional file 1 **Genotyping Conditions. Table S1.** Primers sequences used in iPlex Gold reaction, Sequenom plataform. **Table S2.** Primers and restriction enzymes used in the RFLPs procedures. Click here for file
