# Supplementary material for: Genetic variation at the CYP2C19 gene associated with metabolic syndrome susceptibility in a South Portuguese population: results from the pilot study of the European Health Examination Survey in Portugal
Source: Diabetol Metab Syndr. 2014 Feb 18;6:23. doi: 10.1186/1758-5996-6-23 (PMC3932792; doi:10.1186/1758-5996-6-23)
Supplement: Additional file 1 — Genotyping Conditions. Table S1. Primers sequences used in iPlex Gold reaction, Sequenom plataform. Table S2. Primers and restriction enzymes used in the RFLPs procedures. [file 1758-5996-6-23-S1.docx]

### Additional File 1

### Genotyping Conditions

**Table 1.** Primers sequences used in iPlex Gold reaction, Sequenom Mass Array plataform.

| GENE | NCBI ID | Primer name | Sequence |
| --- | --- | --- | --- |
| *ADCY5* | rs11708067 | Foward | ACGTTGGATGGGACGTGCAAACAGATGTAG |
|  |  | Reverse | ACGTTGGATGTGGATGAAGGCCACTGTATC |
|  |  | Extend | GTGCAAACAGATGTAGATTAATAGAG |
| *ADRB2* | rs1042714 | Foward | ACGTTGGATGCAGGACGATGAGAGACATGA |
|  |  | Reverse | ACGTTGGATGGAAGCCATGCGCCGGACCA |
|  |  | Extend | CCACACCTCGTCCCTTT |
| *ADRB3* | rs4994 | Foward | ACGTTGGATGAACACGTTGGTCATGGTCTG |
|  |  | Reverse | ACGTTGGATGAGGCAACCTGCTGGTCATC |
|  |  | Extend | ATTGCTGGAGTCTCGGAGTCC |
| *APOE* | rs7412 | Foward | ACGTTGGATGTCCGCGATGCCGATGACCT |
|  |  | Reverse | ACGTTGGATGCTCGCGGATGGCGCTGAGG |
|  |  | Extend | TGCCGATGACCTGCAGAAG |
| *CDKAL1* | rs7754840 | Foward | ACGTTGGATGTAACAGAGACATCACTGTCC |
|  |  | Reverse | ACGTTGGATGTGCTGTTGGGGAAGAAGTAG |
|  |  | Extend | AAATCCTCTATCAAGTCAAC |
| *CDKN2A/B* | rs10811661 | Foward | ACGTTGGATGATAAGCGTTCTTGCCCTGTC |
|  |  | Reverse | ACGTTGGATGAGATCAGGAGGGTAATAGAC |
|  |  | Extend | GCCTCCAGCTTTAGTTTTC |
| *CYP2C19* | rs4244285 | Foward | ACGTTGGATGCACTTTCCATAAAAGCAAGG |
|  |  | Reverse | ACGTTGGATGGCAATAATTTTCCCACTATC |
|  |  | Extend | AAGTAATTTGTTATGGGTTCC |
|  |  |  |  |
| *FTO* | rs9939609 | Foward | ACGTTGGATGGTAACAGAGACTATCCAAGTG |
|  |  | Reverse | ACGTTGGATGACACTAACATCAGTTATGC |
|  |  | Extend | CCCCAGACTATCCAAGTGCATCAC |
| *GABRA2* | rs279871 | Foward | ACGTTGGATGGCTATGCTAAGGAGGCTTTT |
|  |  | Reverse | ACGTTGGATGGGGATCAGAGGTAGAACAAA |
|  |  | Extend | CTTTCCTGACATGTATGTGATATATT |
|  |  |  |  |
| *GNPDA2* | rs10938397 | Foward | ACGTTGGATGCGATAATAATGCTAAGAAC |
|  |  | Reverse | ACGTTGGATGCATTAGTATTGTACACACACC |
|  |  | Extend | GGGGCTAAGAACATTCTTGAAAAC |
| *HHEX* | rs1111875 | Foward | ACGTTGGATGAACTTCTCACTCCCTTCCAC |
|  |  | Reverse | ACGTTGGATGAAAAAATGGACCCTGAGTGC |
|  |  | Extend | TTACCATCAAGTCATTTCCTCT |
| *IGF2BP2* | rs4402960 | Foward | ACGTTGGATGTCTATGGAGTTTTGGCCCTG |
|  |  | Reverse | ACGTTGGATGGGGCATGTTTGCAAACACAA |
|  |  | Extend | TTAAGGTAGGATGGACAGTAGATT |
|  |  |  |  |
| *IL6* | rs1800795 | Foward | ACGTTGGATGGATTGTGCAATGTGACGTCC |
|  |  | Reverse | ACGTTGGATGAGCCTCAATGACGACCTAAG |
|  |  | Extend | AATGTGACGTCCTTTAGCAT |
| *KCNJ11* | rs5219 | Foward | ACGTTGGATGTCCGCTGGCGGGCACGGTA |
|  |  | Reverse | ACGTTGGATGGGCATCATCCCCGAGGAATA |
|  |  | Extend | GGGCACGGTACCTGGGCT |

**Table 1.** Primers sequences used in iPlex Gold reaction, Sequenom Mass Array plataform (Continuation).

| GENE | NCBI ID | Primer name | Sequence |
| --- | --- | --- | --- |
| *KCNQ1* | rs2237892 | Foward | ACGTTGGATGCTGGCATGAGCCAGATGATG |
|  |  | Reverse | ACGTTGGATGATCTGGTGGAGAGGGGTTTC |
|  |  | Extend | ACACAGGACTTTGCCACC |
| *KCNQ1* | rs231362 | Foward | ACGTTGGATGGTGTAGCTCACCTGCCTTTG |
|  |  | Reverse | ACGTTGGATGATGGGTTGCCTAGAGACAAG |
|  |  | Extend | TGCCTTTGACCCTGCAC |
| *LDLR* | rs2228671 | Foward | ACGTTGGATGCTCCTTTTCCTCTCTCTCAG |
|  |  | Reverse | ACGTTGGATGTGTAGGAGATGCATTTCCCG |
|  |  | Extend | TCAGTGGGCGACAGATG |
| *MTCH2* | rs10838738 | Foward | ACGTTGGATGCTGTAATGGGCAGGTCATTC |
|  |  | Reverse | ACGTTGGATGGGGTTCTAAAAACTGTTCC |
|  |  | Extend | GACATAATTACCTCATGCAC |
| *MTNR1B* | rs10830963 | Foward | ACGTTGGATGGGCAGAATATTCCCATCAGG |
|  |  | Reverse | ACGTTGGATGCCCCCAGTGATGCTAAGAAT |
|  |  | Extend | GCAGTTACTGGTTCTGGATAG |
| *NOS1AP* | rs12143842 | Foward | ACGTTGGATGTTAGCACCCAGGGTCACATC |
|  |  | Reverse | ACGTTGGATGGCTAGAGAGGCCTTCAACAG |
|  |  | Extend | TTAGCACCCAGGGTCACATCCCAGTT |
| *NOS3* | rs1799983 | Foward | ACGTTGGATGACGGCTGGACCCCAGGAAA |
|  |  | Reverse | ACGTTGGATGGGGGCAGAAGGAAGAGTTC |
|  |  | Extend | CTGCAGGCCCCAGATGA |
|  |  |  |  |
| *NPC1* | rs1805081 | Foward | ACGTTGGATGCTCAACACAATTCCTTTCTG |
|  |  | Reverse | ACGTTGGATGATCCACAGACTCGTCACAGC |
|  |  | Extend | CTTTCTGTAGATTTTCCAGTCC |
| *NPY* | rs16147 | Foward | ACGTTGGATGGATTCTTGTCTCCTGCCAAC |
|  |  | Reverse | ACGTTGGATGCTAGTCGTGGAGATGCCCC |
|  |  | Extend | CTGCCAACAGGACTACCA |
| *PPARG* | rs1801282 | Foward | ACGTTGGATGGTTATGGGTGAAACTCTGGG |
|  |  | Reverse | ACGTTGGATGGTTTGCAGACAGTGTATCAG |
|  |  | Extend | GGGAGATTCTCCTATTGAC |
|  |  |  |  |
| *PTER* | rs10508503 | Foward | ACGTTGGATGGCATTAGTGATTTTGCAGTC |
|  |  | Reverse | ACGTTGGATGATGGCACTGGACAGTTCTGG |
|  |  | Extend | CGTCTATTATGCATCACG |
|  |  |  |  |
| *SH2B1* | rs7498665 | Foward | ACGTTGGATGCGCATCCCCATTGAAGAGG |
|  |  | Reverse | ACGTTGGATGTGTTTCCGGAGTGTCCAAGG |
|  |  | Extend | ATTGAAGAGGGACCCCCA |
| *SLC30A8* | rs13266634 | Foward | ACGTTGGATGTCTCCCTGTGCTTCTTTATC |
|  |  | Reverse | ACGTTGGATGGCAATTTCTCTCCGAACCAC |
|  |  | Extend | TATCAACAGCAGCCAGC |
| *TCF7L2* | rs7903146 | Foward | ACGTTGGATGACAATTAGAGAGCTAAGCAC |
|  |  | Reverse | ACGTTGGATGCGTCTGAAAACTAAGGGTGC |
|  |  | Extend | GAGCTAAGCACTTTTTAGATA |
| *TMEM18* | rs6548238 | Foward | ACGTTGGATGAAAGAGACAGGAGAAGGGAG |
|  |  | Reverse | ACGTTGGATGAATAGGCCCCAGCATAAGTC |
|  |  | Extend | CAGCTGGGAGCACAGGGA |
| *TPMT* | rs1142345 | Foward | ACGTTGGATGCCTCAAAAACATGTCAGTGTG |
|  |  | Reverse | ACGTTGGATGGGGAATTGACTGTCTTTTTG |
|  |  | Extend | GTCTCATTTACTTTTCTGTAAGTAGA |

**Table 2.** Primers and restriction enzymes used in the RFLPs method.

| Gene | NCBI ID | Primer name | Sequence | Restriction Enzyme |
| --- | --- | --- | --- | --- |
| *ACE* | rs4646994 | Foward | CTGGAGACCACTCCCATCCTTTCT | ^1^ |
|  |  | Reverse | GATGTGGCCATCACATTCGTCAGAT |  |
| *ADRB1* | rs1801252 | Foward | CCGGGCTTCTGGGGTGTTCC | Eco0109I |
|  |  | Reverse | GGCGAGGTGATGGCGAGGTAGC |  |
| *ADRB2* | rs1042713 | Foward | CTTCTTGCTGGCACGCAAT | BsrDI |
|  |  | Reverse | CCAGTGAAGTGATGAAGTAGTTGG |  |
| *CYP2C8* | rs10509681 | Foward | CTGCTGAGAAAGGCATGAAG | XmnI |
|  |  | Reverse | CTTCCGTGCTACATGATGACG |  |
| *CYP2D6* | rs16947 | Foward | GCTGGGGCCTGAGACTT | HhaI |
|  |  | Reverse | GGCTATCACCAGGGGCTGGTGCT |  |
|  |  |  |  |  |
| *NOS3* | rs2070744 | Foward | TGGAGAGTGCTGGTGTACCCCA | HpaII |
|  |  | Reverse | GCCTCCACCCCCACCCTGTC |  |
|  |  |  |  |  |
| *CYP2C9* | rs1799853 | Foward | CACTGGCTGAAAGAGCTAACAGAG | AvaII |
|  |  | Reverse | GTGATATGGAGTAGGGTCACCCAC |  |

^1^It was detected by fragment different size because this polymorphism is an insertion/delection of 289 pb.
